# Social Determinants of Health in the COVID-19 Pandemic Context of the Migrant Population Living in Settlements in Spain

**DOI:** 10.3389/ijph.2022.1604628

**Published:** 2022-08-04

**Authors:** Regina Allande-Cussó, Juan Jesús García-Iglesias, Rosario Miranda-Plata, Rocío Pichardo-Hexamer, Carlos Ruiz-Frutos, Juan Gómez-Salgado

**Affiliations:** ^1^ Department of Nursing, Faculty of Nursing, Podiatry and Physiotherapy, University of Seville, Seville, Spain; ^2^ Department of Sociology, Social Work and Public Health, Faculty of Labour Sciences, University of Huelva, Huelva, Spain; ^3^ Spanish Red Cross of Huelva, Huelva, Spain; ^4^ Safety and Health Postgraduate Programme, Universidad Espíritu Santo, Guayaquil, Ecuador

**Keywords:** public health, COVID-19, social determinants of health, migrants, psychological distress, fear, settlements, work engagement

## Abstract

**Objectives:** The aim of this study was to describe and evaluate the sociodemographic profile and assess the levels of anxiety and fear, work engagement, and psychological distress on a sample of migrants living in settlements in the province of Huelva (Spain) during the COVID-19 pandemic.

**Methods:** A descriptive cross-sectional study was conducted on a sample of 623 migrants during the months of April to June 2021, based on the Anxiety and Fear of COVID-19 (AMICO) assessment scale, the 9-item Utrecht Work Engagement Scale to assess work engagement, and the General Health Questionnaire (GHQ-12) to analyse psychological distress.

**Results:** A low level of education, dwelling of less than 3 m^2^ and the desire to return to the country of origin may be related to the presence of anxiety and fear of COVID-19 and lower levels of work engagement.

**Conclusion:** There is a need to improve the study of the concept of health of the migrant population residing in the settlements of Huelva (Spain) and the assessment of their physical and mental health, in an official way.

## Introduction

COVID-19 has brought with it a number of social, economic and political consequences in all countries worldwide, while it is true that each country has shown a different impact on this issue [1]. In 2015, the United Nations General Assembly developed the 2030 Agenda for Sustainable Development, where one of its goals is to make cities and human settlements inclusive, safe, resilient, and sustainable, but the reality is quite different [2]. Globally, it is estimated that 1 in 4 people live in slums and informal settlements [3]. In Spain, the National Institute of Statistics estimates that 19.7% of the population living in Spain does so in substandard housing, especially in the south of the country [4].

In this sense, people living in informal settlements tend to have poor living conditions, with small dwellings in close proximity to each other and made of cheap and/or recycled building materials, and are often overcrowded due to the large number of dwellers [5], making it more difficult to comply with preventive measures against COVID-19, thus increasing the risk of COVID-19 transmission [6].

On the other hand, WHO defines the concept of social determinants of health (SDOH) as the social, physical, and economic conditions that impact health, both physically and mentally [7]. People living in informal settlements often have limited or no income, and a large proportion of them work in sectors such as agriculture, cleaning, transport, etc., and are therefore often employed in low-skilled, low-paid, temporary jobs with a higher risk of exposure to the possible spread of COVID-19 [8]. To this difficulty it may be added the fear they may have of losing their job, which is their main livelihood [9, 10], together with the uncertainty that COVID-19 may significantly affect the physical and mental health of the population in general, and of people living in poorer areas in particular [11]. In the face of this circumstance, both work demands and work loads, and even job opportunities, have changed, especially in a vulnerable group such as the migrants one. Interest to assess work engagement, together with emotional distress [12] and the presence of anxiety and fear of COVID-19 has then risen, given their intrinsic relationship with the social determinants of health [13] and due to the impact that the pandemic has on these determinants for this population group which is at risk of social exclusion [14].

Therefore, the aim of this study was to describe and analyse the sociodemographic profile and to assess the levels of anxiety and fear, work engagement, and psychological distress intrinsically related to the social determinants of health, on a sample of migrants living in settlements in the province of Huelva (Spain) during the COVID-19 pandemic, as a starting point for the detection of specific socio-health interventions in the pandemic context.

## Methods

### Study Design

A descriptive, cross-sectional, questionnaire-based study was conducted from April to June 2021.

### Population and Sample

The province of Huelva is located in the southwest of Spain and has a surface area of 10,128 km^2^ and a population of just over half a million, of which 48,304 are foreigners according to the Spanish National Statistics Institute [15]. Its economy is mainly based on the agricultural, service, and industrial sectors. Job opportunities in the agricultural sector have led many people, especially from North Africa, to migrate to Huelva on a temporary basis to work in the intensive agricultural campaigns to harvest red fruits. Others, however, tend to work in other professions in the primary sector or are unemployed [16]. Since there is no real record of the total number of subjects residing in the settlements in the province of Huelva (southern Spain), the necessary sample size could not be estimated. However, the Red Cross Organisation, a participating entity in this study, estimated a population size of approximately 5,000 subjects. With this, a sample of at least 260 subjects was estimated, considering a confidence level of 95%, precision of 3%, a proportion of 5%, and an expected proportion of losses of 25%. However, the final sample consisted of 623 migrants, regardless of age, who voluntarily agreed to participate in the study.

The sample was selected by non-probability snowball sampling. An online questionnaire was created with the Google Forms^©^ application, containing information about the study and informed consent, as well as other items related to the study variables. This questionnaire was also translated into French and Arabic by two professional cultural mediators from the Red Cross Organisation, with degrees in French and Arabic Studies, respectively. The final tool was administered in the settlements, with the help of Red Cross staff and volunteers. All collaborators carried out the data collection using the link to the questionnaire, in the language of the interviewee, using tablets or mobile phones of the organisation itself.

All subjects were informed of the purpose of the study and the possibility of participating in it on a voluntary, anonymous, and confidential basis. In addition, all subjects were required to give their consent, which was recorded by the interviewer collaborator by ticking a specific box before the start of the survey.

### Variables

The present study included socio-demographic variables (sex, age, settlement, country of origin, work activity, highest level of education, work permit, housing situation), variables related to health and the COVID-19 pandemic (positive diagnosis, isolation, chronic diseases, consumption, hospitalisation, preventive measures, and self-perception of health), and those of the measuring instruments themselves.

### Instrument

The level of anxiety and fear of COVID-19 was assessed using the “Assessment of Anxiety and Fear of COVID-19” (AMICO) scale, validated by Gómez-Salgado et al. [17]. This scale consists of 2 dimensions (anxiety and fear), and 16 items, which are scored from 0 to 10 points. The final score is obtained by calculating the mean score the subject gives to each item, and the cut-off point is set at 6.4 points, indicating that scores higher than this represent high levels of anxiety and fear of COVID-19. After the validation process, the scale obtained a Cronbach’s alpha of 0.92. The scale obtained convergent validity, a sensitivity value of 90.48%, and a specificity value of 76% [17, 18]. In addition, a process of cultural adaptation of the scale into French and Arabic was carried out based on the principles of good practice for the translation and cultural adaptation process for patient-reported outcomes measures [19]. The process was carried out in three phases: translation of the AMICO scale into both languages by two native male key informants identified by the Red Cross organisation from among the people living in the settlements, with a minimum educational level of secondary school education; back-translation of both versions into Spanish by two professional women, cultural mediators from the Red Cross organisation, with degrees in French and Arabic Studies; face validity analysis of the versions translated into English and Arabic with respect to the original Spanish version, in which all the translators and back-translators participated, as well as the team of authors of this study and of the AMICO scale.

Work engagement was assessed using the 9-item Utrecht Work Engagement Scale (UWES) questionnaire, with a Likert-type response scale between 0 (never) and 5 (always) [20]. The tool assesses work engagement on the basis of three independent but related dimensions or subscales (vigour, dedication, and absorption). Its Spanish validation obtained a Cronbach’s alpha value of 0.9, and between 0.79 and 0.84 for the 3 subscales, and the final score of the scale is obtained by calculating the average of the scores. The values of the means scores of UWES are: very low 0 to 0.99; low 1 to 1.99; medium 2 to 3.99; high 4 to 4.99; very high 5 to 6 [20]. The UWES-9 version validated in French obtained a Cronbach’s alpha value of 0.92, and between 0.80 and 0.90 for the 3 subscales [21], and the validated Arabic version obtained a Cronbach’s alpha value of 0.91, and between 0.85 and 0.91 for the 3 subscales [22].

Psychological distress was measured using the General Health Questionnaire (GHQ-12), a psychometric instrument widely used as a screening tool for non-psychotic psychiatric disorders [23]. It consists of 12 items with four response options, and each item can obtain a score of 0–2 points, to result in a total score ranging from 0 to 24. This questionnaire, developed by Goldberg, has been translated and validated in many countries, presenting Cronbach’s alpha values of 0.82–0.86 [23], also showing good reliability in its Spanish-speaking version with 0.86 and 0.76 in the Spanish population [24]. The GHQ-12 scale validated in French obtained a Cronbach’s alpha value of 0.92 [25], and the validated Arabic version obtained a Cronbach’s alpha value of 0.86 [26]. For this study, the global score was used as a single factor whose reliability, estimated by Cronbach’s alpha, was *α* = 0.851. The cut-off point established for the general population was 12, with subjects whose scores greater than or equal to 12 being considered a potential case of psychiatric morbidity [24].

### Data Analysis

Univariate and bivariate descriptive data analysis was performed using IBM SPSS Software^©^ v26 [27]. The raw data were recorded in a spreadsheet, and subsequently transferred to the SPSS^©^ software in a rigorous manner. After that, the database was revised, naming each study variable and defining its characteristics, in order to obtain a final refined database. The Kolmogorov-Smirnov test showed that the data distribution regarding each of the measurement scales used was normal (*p* value ≥ 0.2), so parametric statistics were used. For data analysis, descriptive statistics of central tendency and dispersion were developed as well as contrast tests, previously studying the homogeneity of variances, such as T-Student, one-factor ANOVA, and Pearson’s correlation. In the case of one-factor ANOVA the existence of homogeneity of variances was previously analysed when the *p*-value of Levene’s statistic was greater than 0.05. A confidence level of 95% was established for all the statistical tests used.

To establish the relationship between work engagement (total score of the UWES-9 questionnaire) and the level of anxiety and fear of COVID-19 (total score of the AMICO scale), and the rest of the variables, a categorical regression analysis (CATREG) was performed, since these variables are qualitative in nature. The CATREG analysis includes characteristic aspects of classical regression analysis: coefficient of determination (R2), analysis of the variance in the regression, and significance of the model parameters. For its calculation, the optimal scaling option was selected in SPSS^©^ software, and dummy variables were introduced for the calculation of the final model [27].

### Ethical Issues

Permission was obtained from the Research Ethics Committee of the Regional Government of Andalusia (Ref. 1539-N-20). In addition, all sample subjects gave their consent voluntarily, having been informed of the purpose of the study, ensuring at all times the confidentiality of the data and the anonymity of the participants, based on Organic Law 3/2018, of 5 December, on Personal Data Protection and Guarantee of Digital Rights [28].

## Results

### Univariate Analysis

The socio-demographic profile of the sample is described in Table 1. Of the total 623 subjects making up the sample, 84% were male and the mean age was 33.4 years (SD = 10.7 years). Regarding the level of education, 81.2% had no minimum education, 18% had primary or secondary education, and 0.8% had completed higher education. On the other hand, 11.7% worked on a continuous basis, 23% only on specific days, and 65.2% reported that they were not working. In this respect, only 39.3% had a valid work permit in Spain. Among them, the highest percentages are Morocco (27.93%), Mali (27.29%), and Ghana (21.19%). The remaining 23.59% come from other countries of the African continent such as Sahara, Mauritania, Senegal, or Algeria.

**TABLE 1:** Description of the sample profile (Social determinants of health and migrant population in Covid-19 pandemic, Spain, 2021).

Sex	Male	*n* = 524 (84%)	Total sample *N* = 623
	Female	*n* = 99 (16%)	
Age	Mean	33.4	
	Standard deviation	10.7	
Nationality	Marocco	*n* = 174 (27.93%)	
	Mali	*n* = 170 (27.29%)	
	Ghana	*n* = 132 (21.19%)	
	Other countries from Africa	*n* = 147 (23,59%)	
Educational level	None/Primary studies	*n* = 506 (81.2%)	
	Secondary studies	*n* = 113 (18%)	
	Higher studies	*n* = 4 (0.8%)	
Work activity	Yes, on a continued basis	*n* = 73 (11.7%)	
	Yes, on specific days	*n* = 143 (23%)	
	No	*n* = 407 (65.2%)	
Work permit	Yes	*n* = 245 (39.3%)	
	In process	*n* = 18 (2.9%)	
	No	*n* = 360 (57.8%)	
Arrival to Spain	By sea: dinghy	*n* = 375 (60.1%)	
	By land	*n* = 227 (36.4%)	
	Other ways, not specified	*n* = 21 (3.5%)	
Desire to return	No	*n* = 480 (77%)	
	Yes	*n* = 143 (23%)	
Dwelling	Less than 3 m^2^	*n* = 103 (16.5%)	
	More than 3 m^2^	*n* = 520 (83.5%)	
AMICO scale	Mean	6.5	Maximum score = 10 points
	Standard deviation	2.4	
UWES-9 scale	Mean	5.38	Maximum score = 6 points
	Standard deviation	1.38	
GHQ-12 questionnaire	Mean	14.8	Maximum score = 48 points
	Standard deviation	4.8	

In relation to the migration process, 60.1% indicated that they had entered the country by sea, and 77% of the total sample did not wish to return to their country of origin. Regarding the dwelling they occupied at the time of data collection, 16.3% claimed to live in an area of less than 3 m^2^.

Regarding the distribution of the sample subjects by the different settlements in the province of Huelva (southern Spain), 26% resided in the settlement of Lepe, 22% in Las Sevillanas, 18% in Las Madres, 13% in Los Gagos, 3.8% in Valdefresas, and the rest of the subjects were distributed in smaller percentages in up to 8 smaller settlements.

Considering health-related variables described in Table 2, 96.5% said they did not have any chronic diseases, and 79,2% did not consume any toxic substances. However, 14.4% were active smokers, and 6.4% consumed alcohol on a daily basis. Also, the mean score obtained for self-perceived health status was 8.74 (SD = 1.2), out of a total score of 10. Regarding the questions related to the COVID-19 pandemic, only 3.1% had been infected with COVID-19, although 8.1% had experienced confinement for being in close contact with a positive person. 46.5% of the sample reported not having received any training in pandemic prevention and control measures, and only 34.4% of the sample who were working had received protective equipment from their employers during their employment.

**TABLE 2:** Description of health variables Description of the sample profile (Social determinants of health and migrant population in Covid-19 pandemic, Spain, 2021).

Chronic diseases	Yes	*n* = 22 (3.5%)	Total sample *N* = 623
	No	*n* = 601 (96.5%)	
Toxic substance consumption	Tobacco	*n* = 90 (14.4%)	
	Alcohol	*n* = 40 (6.4%)	
	None	*n* = 493 (79.2%)	
COVID-19 infection	Yes	*n* = 20 (3.3%)	
	No	*n* = 603 (96.7%)	
Confinement	Yes	*n* = 51 (8.1%)	
	No	*n* = 572 (91.9%)	
Training in safety	Yes	*n* = 333 (53.4%)	
	No	*n* = 290 (46.6%)	
Protective equipment	Yes	*n* = 214 (34.4%)	
	No	*n* = 409 (65.6.6%)	
Self-perceived health	Mean	8.74	Maximum score = 10 points
	Standard deviation	1.2	

The mean work engagement score was 5.38 points (SD = 1.38), indicating a very high overall level. (Table 1). For the vigour and dedication dimensions, mean scores of 5.60 (SD = 1.4) and 5.37 (SD = 1.5) were obtained, respectively, both being categorised at the high level. In the same line, the mean score for the absorption dimension was 5.59 (SD = 1.6), which is interpreted as a very high level.

Regarding the level of perceived anxiety and fear, the mean score of the AMICO scale was 6.54 points (SD = 5.4). This score was higher than 6.4, indicating the presence of both constructs in the study sample.

Likewise, the mean score of the GHQ questionnaire was 14.12 points (SD = 4.8), and this would indicate the possibility that the sample is suffering from psychological distress.

### Bivariate Analysis

#### Level of Anxiety and Fear

No significant differences were found between the level of anxiety and fear, which were assessed by the AMICO scale, and the different values of the work activity, work permit, route of entry to Spain, and housing variables (Table 3). In relation to the education variable, the differences between the mean scores of the variable were significant (F = 2.3; *p* = 0.045). Thus, subjects with no studies presented higher levels of anxiety than the rest of the categories of the variable. In the same sense, people who wished to stay in Spain presented higher levels of anxiety and fear, and the hypothesis test was significant (*t* = 2.2; *p* = 0.01) (Table 3). Thus, subjects whose response was that they did not wish to return to their country of origin had a mean score on the AMICO scale of 5.45 points, while those subjects who did wish to return to their country of origin had a mean score of 4.74 points.

**TABLE 3:** Results of the hypothesis test Description of the sample profile (Social determinants of health and migrant population in Covid-19 pandemic, Spain, 2021).

Scale	Variable	Variable values	Score	Statistical hypothesis test	*p*-Value
AMICO	Educational level	Secondary studies	5.18	F = 2.3^a^	**0.045** ^**a**^
		Primary studies	5.17		
		Higher studies	4.47		
		No studies	6.03		
	Work activity	No	5.20	F = 0.94^a^	0.07^a^
		Yes, on a continued basis during the campaign	5.31		
		Yes, on specific days	5.49		
	Work permit	In process	4.89	F = 1.000^a^	0.60^a^
		No	5.25		
		Yes	5.36		
	Arrival to Spain	Other	5.24	F = 0.91^a^	0.57^a^
		Dinghy to the coast	5.29		
		By land	8.94		
	Desire to return	No	5.45	t = 2.2^b^	**0.01** ^**b**^
		Yes	4.75		
	Dwelling	More than 3 m/^2^	5.34	t = 1.32^b^	**0.02** ^**b**^
		Less than 3 m/^2^	5.01		
UWES-9	Educational level	Secondary studies	6.06	F = 3.61^a^	**0.01** ^**a**^
		Primary studies	5.06		
		Higher studies	5.56		
		No studies	5.37		
	Work activity	No	5.50	F = 1.6^a^	**0.04** ^**a**^
		Yes, on a continued basis during the campaign	6.16		
		Yes, on specific days	5.24		
	Work permit	In process	4.62	F = 1.22^a^	0.14^a^
		No	5.60		
		Yes	5.61		
	Arrival to Spain	Other	5.73	F = 1.83^a^	0.06^a^
		Dinghy to the coast	5.40		
		By land	5.39		
	Desire to return	No	6.21	t = 4.2^b^	**0.001** ^**b**^
		Yes	5.26		
	Dwelling	More than 3 m/^2^	5.47	t = 2.17^b^	**0.03** ^**b**^
		Less than 3 m/^2^	6.16		
GHQ-12	Educational level	Secondary studies	17	F = 1.44^a^	0.09^a^
		Primary studies	16		
		Higher studies	15		
		No studies	16		
	Work activity	No	17	F = 0.98^a^	0.27^a^
		Yes, on a continued basis during the campaign	12		
		Yes, on specific days	15		
	Work permit	In process	15	F = 1.23^a^	0.15^a^
		No	16		
		Yes	16		
	Arrival to Spain	Other	17	F = 1.05^a^	0.8^a^
		Dinghy to the coast	16		
		By land	18		
	Desire to return	No	16	F = 0.60^b^	0.4^b^
		Yes	15		
	Dwelling	More than 3 m/^2^	16	t = 2.23^b^	**0.02** ^**b**^
		Less than 3 m/^2^	18		

Significant *p*-values in bold.

^a^ANOVA.

^b^T-student.

### Work Engagement

The results of the statistical analysis reported no significant differences between the values of the work permit and route of entry to Spain variables and the total level of work engagement, as assessed by the UWES-9 scale (Table 3).

On the contrary, significant differences were found for the following variables: education (F = 3.61; *p* = 0.01), housing (*t* = 2.17; *p* = 0.03), desire to return to their country of origin (*t* = 4.2; *p* = 0.001), and work activity (F = 1.6; *p* = 0.04). Subjects in the sample who reported not working obtained a mean work engagement score of 5.50 points, and those who worked on a continuous basis obtained a mean work engagement score of 6.16 points. Regarding the education variable, subjects with primary school education had a work engagement level of 5.06 points; on the other hand, subjects with secondary school and higher education had scores of 6.06 and 5.56, respectively. Likewise, those who reported their desire to return to their country of origin obtained a mean score of 6.21 points, as opposed to those who wished to stay in Spain, who obtained a mean of 5.26 points. Similarly, participants whose housing was less than 3 m^2^ showed higher levels of work engagement than those in the opposite situation (6.16 and 5.47, respectively) (Table 3).

### Psychological Distress

No significant differences were found between the presence of psychological distress, as assessed by the GHQ-12 scale, and the different values of the education, work activity, work permit, route of entry to Spain, and desire to return to their country of origin. On the other hand, subjects whose dwelling was smaller than 3 m^2^ scored 18 points, compared to those whose dwelling was larger than 3 m^2^, who scored 16 points. The results of the statistical analysis therefore showed significant differences for this variable (*t* = 2.23; *p* = 0.02) (Table 3).

### Correlation Between Questionnaires

The correlation between the mean score of the GHQ-12 questionnaire and the mean score of the AMICO questionnaire was studied, and although it was significant (*p* = 0.01), it obtained a value of *r* = 0.27, indicating a direct but slight correlation. Likewise, the correlation between the UWES-9 questionnaire and the GHQ-12 questionnaire showed a value of *p* = −0.20, reflecting an indirect but slight correlation. The correlation between the mean score of the UWES-9 questionnaire and the AMICO questionnaire was also significant, with a value of *p* = −041. This indicates an indirect correlation, with a moderate effect.

### Regression Analysis

For the regression analysis, those variables that had obtained significant differences in the hypothesis tests were inserted into the test models. Table 4 shows the results of the categorical regression. It is observed that the lower educational level, dwelling of less than 3 m^2^ and desire to return variables are related to the presence of anxiety and fear of COVID-19, and this model explains 65% of the variation of the variable. Similarly, the desire to stay in Spain, dwelling of less than 3 m^2^, lower educational level, and not having a continuous work activity are related to lower levels of work engagement. In the same sense, dwelling of less than 3 m^2^ is also related to the presence of psychological distress.

**TABLE 4:** Regression model Description of the sample profile (Social determinants of health and migrant population in Covid-19 pandemic, Spain, 2021).

Scale	Variables	BETA coefficient	Variable significance (*p*-value)	*R* ^2^ value	Model significance (*p*-value)
AMICO	Educational level	−0.13	0.03	0.65	0.01^a^
	Desire to return	−0.13	0.02		
	Dwelling	−0.11	0.01		
UWES-9	Educational level	0.11	0.001	0.22	0.001^a^
	Work activity	−023	0.001		
	Desire to return	0.34	0.001		
	Dwelling	0.21	0.01		
GHQ-12	Dwelling	0.31	0.005	0.61	0.06^a^

a Anova.

## Discussion

The aim of this study was to assess emotional distress and work engagement in the migrant population residing in the settlements of the province of Huelva (Spain) during the COVID-19 pandemic. People living in informal settlements present anxiety and fear of contagion of COVID-19 and the possibility of becoming unemployed.

It is paradoxical that people with poor housing conditions, many of whom are unemployed, report good self-perceived health (8.74 out of 10), although this phenomenon could be explained by the “healthy migrant” theory proposed by Lu in 2008 [29], and corroborated in subsequent studies such as the one conducted by De Wet et al. in Johannesburg (South Africa) [30]. This theory attempts to explain this “epidemiological paradox” in which migrants, especially younger people, are generally healthier than populations in recipient societies [31].

In relation to the questions related to the COVID-19 pandemic, only 3.1% had suffered COVID-19. This could be justified by the high unemployment rate of the sample at the time of data collection and the low incidence of the disease in the study area (Huelva), even though only one in three people had received personal protective equipment at work and reported gaps in knowledge, in line with a study of food handlers in an informal urban settlement in Nairobi (Kenya) during COVID-19 [32]. Similar studies indicate that when a person in an informal neighbourhood leaves the neighbourhood, it may be either to go to work, to look for work, or to look for resources such as food, water, clothing, among others [33]. Keeping in mind that the data were collected between April and June 2021, the red fruit harvesting season was coming to an end, which would justify the high rate of unemployment and the low percentage of infections, yet being well below those found in similar samples [29, 31].

Regarding the level of perceived anxiety and fear, the mean score of the AMICO scale was above the cut-off point (>6.4 points), indicating the presence of both constructs in the study sample. These results are similar to those found in samples from informal settlements in Peru through the Fear of COVID-19 Scale [34], where four out of six offered scores above the cut-off point in Peru [35]. In contrast, the prevalence of depression and anxiety during the COVID-19 pandemic among residents of an urban slum in northern India yielded values below those found in our sample [36]. However, no significant differences were found between the level of anxiety and fear, as assessed by the AMICO scale, and the different values of the work activity, work permit, route of entry to Spain, and dwelling variables. But statistically significant differences were found between those subjects with no education and those who wished to stay in Spain. In comparison with data from the general Spanish population assessed by the AMICO scale [37], the sample of residents of informal settlements showed higher levels of fear and anxiety (6.54 points; SD = 5.4), as compared to the general Spanish population (5.54 points; SD = 1.83). Perhaps, lack of knowledge about the disease, fear of unemployment, and lack of resources to meet basic needs may be behind these differences [38].

Like any research study, the present one has several limitations. Firstly, due to its nature as a descriptive cross-sectional study, it does not allow to draw cause and effect conclusions. In addition, the timing of data collection may also influence the response of individuals in such changing circumstances. Secondly, self-reporting and social desirability bias are inherent in any study based on questionnaires, although in the specific case of the data collected on contagion and close contacts with other people infected with COVID-19 it is particularly relevant because there is likely to be an under-reporting of these cases against the self-reported reality, as well as fear of job loss, even if it is precarious. However, the present study provides the opportunity to describe for the first time the sociodemographic characteristics and health beliefs of the migrant population living in settlements in Huelva (Spain); with this, this study is also positioned as a starting point for the design of specific intervention strategies that should be aimed at improving living and working conditions, intrinsically linked to the social determinants of health and the improvement of public health in Spain.

### Conclusion

A low level of education, dwelling of less than 3 m^2^ and the desire to return to the country of origin may be related to the presence of anxiety and fear of COVID-19. Moreover, the desire to stay in Spain and not having a continuous work activity are related to lower levels of work engagement. On the other hand, there is a need to improve the study of the concept of health of the migrant population residing in the settlements of Huelva (Spain) and the assessment of their physical and mental health, as well as social, economic conditions the impact of the COVID-19 pandemic on this population, in an official way.

## References

[1] Chakraborty I Maity P De Oliveira Andrade R Arndt C Davies R Gabriel S COVID-19 Outbreak: Migration, Effects on Society, Global Environment and Prevention. Sci Total Environ (2020) 728(January):138882. 10.1016/j.scitotenv.2020.138882 32335410 PMC7175860

[2] United Nations. The Sustainable Development Agenda. [Internet]. San Francisco-USA: United Nations (2015). Available from: https://unstats.un.org/sdgs/report/2016/goal-11 .

[3] Lilford RJ Oyebode O Satterthwaite D Melendez-Torres GJ Chen YF Mberu B Improving the Health and Welfare of People Who Live in Slums. Lancet (2017) 389(10068):559–70. 10.1016/S0140-6736(16)31848-7 27760702

[4] Instituto Nacional de Estadística. Objetivo 11. Lograr Que Las Ciudades Y Los Asentamientos Humanos Sean Inclusivos, Seguros, Resilientes Y Sostenibles. Indicadores de la Agenda 2030 para el Desarrollo Sostenible (2021). Available from: https://www.ine.es/dyngs/ODS/es/indicador.htm?id=4909#!subGraph71_175 (Accessed November 1, 2021).

[5] Bhardwaj G Esch T Lall S Marconcini M Soppelsa M Wahba S. Cities, Crowding, and the Coronavirus: Predicting Contagion Risk Hotspots. Washington, DC: World Bank (2020). Available from: https://openknowledge.worldbank.org/handle/10986/33648 (Accessed November 1, 2021).

[6] Nyashanu M Simbanegavi P Gibson L. Exploring the Impact of COVID-19 Pandemic Lockdown on Informal Settlements in Tshwane Gauteng Province, South Africa. Glob Public Health (2020) 15(10):1443–53. 10.1080/17441692.2020.1805787 32780633

[7] World Health Organization. Social Determinants of Health. [Internet] (2022). Available from: https://www.who.int/teams/social-determinants-of-health (Accessed June 15, 2022).

[8] Koh D. Migrant Workers and COVID-19. Occup Environ Med (2020) 77(9):634–6. 10.1136/oemed-2020-106626 32513832 PMC7476302

[9] Kawohl W Nordt C. COVID-19, Unemployment, and Suicide. Lancet Psychiatry (2020) 7:389–90. 10.1016/S2215-0366(20)30141-3 32353269 PMC7185950

[10] Arndt C Davies R Gabriel S Harris L Makrelov K Robinson S Covid-19 Lockdowns, Income Distribution, and Food Security: An Analysis for South Africa. Glob Food Sec (2020) 26:100410. 10.1016/j.gfs.2020.100410 32834955 PMC7366977

[11] Ornell F Schuch JB Sordi AO Kessler FHP. ‘“Pandemic Fear”’ and COVID-19: Mental Health burden and Strategies. Braz J Psychiatry (2020) 42(3):232–5. 10.1590/1516-4446-2020-0008 32267343 PMC7236170

[12] Bakker AB Demerouti E. The Job Demands-Resources Model: State of the Art. J Manag Psychol (2007) 22(3):309–28. 10.1108/0268394071073311

[13] Braveman P Egerter S Williams DR. The Social Determinants of Health: Coming of Age. Annu Rev Public Health (2011) 32:381–98. 10.1146/annurev-publhealth-031210-101218 21091195

[14] Dalsania AK Fastiggi MJ Kahlam A Shah R Patel K Shiau S The Relationship between Social Determinants of Health and Racial Disparities in COVID-19 Mortality. J Racial Ethn Health Disparities (2022) 9(1):288–95. 10.1007/s40615-020-00952-y 33403652 PMC7785288

[15] Economy Department of Spanish Goverment. National Institute of Statistics. [Internet]. Madrid (2022). Available from: https://www.ine.es/jaxiT3/Tabla.htm?t=31304 (Accessed November 1, 2021).

[16] Morán M. Realidad de los asentamientos en la provincia de Huelva. [Internet]. Huelva (2018). Available from: https://www.apdha.org/media/Informe-Asentamientos-Mesa-Integracion-Huelva.pdf (Accessed November 1, 2021).

[17] Gómez-Salgado J Allande-Cussó R Domínguez S García-Iglesias JJ Coronado-Vázquez V Ruiz-Frutos C Design of Fear and Anxiety of COVID-19 Assessment Tool in Spanish Adult Population. Brain Sci (2021) 11(3):328. 10.3390/brainsci11030328 33807643 PMC8001709

[18] Gómez-Salgado J Allande-Cussó R Rodríguez-Domínguez C Domínguez-Salas S Camacho-Martín S Romero-Ruiz A Development and Criterion Validity of the Covid-19 Anxiety and Fear Assessment Scale for the Adult Population: A Psychometric Study. Sicience Prog (2021) 104:368504211050291. 10.1177/00368504211050291 PMC10306143 34698544

[19] Wild D Grove A Martin M Eremenco S McElroy S Verjee-Lorenz A Principles of Good Practice for the Translation and Cultural Adaptation Process for Patient-Reported Outcomes (PRO) Measures: Report of the ISPOR Task Force for Translation and Cultural Adaptation. Value Health (2005) 8(2):94–104. 10.1111/j.1524-4733.2005.04054.x 15804318

[20] Schaufeli W Salanova M González-romá V Bakker A. The Measurement of Engagement and Burnout: A Two Sample Confirmatory Factor Analytic Approach. J Happiness Stud (2002) 3(1):71–92.

[21] Zecca G Györkös C Becker J Massoudi K De Bruin GP Rossier J Validation of the French Utrecht Work Engagement Scale and its Relationship with Personality Traits and Impulsivity. Eur Rev Appl Psychol (2015) 65(1):19–28. 10.1016/j.erap.2014.10.003

[22] Eman-Nafa A. Psychometrics of Arabic Version of the UWES-9. Int J Appl Or Innov Eng Manag (2016) 5(9):191–6. Available from: http://www.ijaiem.org/Volume5Issue9/IJAIEM-2016-09-28-40.pdf .

[23] Goldberg D Gater R Sartorius N Üstün T Piccinelli M Gureje O The Validity of Two Versions of the GHQ in the WHO Study of Mental Illness in General Health Care. Psychol Med (1997) 27:191–7. 10.1017/s0033291796004242 9122299

[24] Rocha K Pérez K Rodríguez M Borrell C Obiols J. Propiedades psicométricas y valores normativos del General Health Questionnaire (GHQ-12) enpoblación general española. Nternational J Clin Heal Psychol (2011) 11(1):125–39.

[25] Lesage F-X Martens-Resende S Deschamps F Berjot S. Validation of the General *Health Questionnaire* (GHQ-12) Adapted to a Work-Related Context. Open J Prev Med (2011) 01(02):44–8. 10.4236/ojpm.2011.12007

[26] Daradkeh T Ghubash R El-Rufaie O. Reliability, Validity, and Factor Structure of the Arabic Version of the 12-item General Health Questionnaire. Psychol Rep (2001) 89:85–94. 10.2466/pr0.2001.89.1.85 11729557

[27] IBM Corporation. IBM SPSS Statistics for Windows, Version 28.0. Armonk, NY: IBM Corporation (2021).

[28] Spanish Government. Ley Orgánica 3/2018, de 5 de diciembre, de Protección de Datos Personales y garantía de los derechos digitales. Madrid: Spanish Government (2018). Available from: https://www.boe.es/eli/es/lo/2018/12/05/3/con (Accessed November 10, 2021).

[29] Lu Y. Test of the “Healthy Migrant Hypothesis”: A Longitudinal Analysis of Health Selectivity of Internal Migration in Indonesia. Soc Sci Med (2008) 67(8):1331–9. 10.1016/j.socscimed.2008.06.017 18639967

[30] De Wet T Plagerson S Harpham T Mathee A. Poor Housing, Good Health: A Comparison of Formal and Informal Housing in Johannesburg, South Africa. Int J Public Health (2011) 56(6):625–33. 10.1007/s00038-011-0269-1 21691863

[31] Seidlein L Alabaster G Deen J Knudsen J. Crowding Has Consequences: Prevention and Management of COVID-19 in Informal Urban Settlements. Build Environ (2020) 188(January):107472. 10.1016/j.buildenv.2020.107472 33250561 PMC7680649

[32] Wainaina E Otieno CA Kamau J Nyachieo A Lowther SA. Norovirus Infections and Knowledge, Attitudes and Practices in Food Safety Among Food Handlers in an Informal Urban Settlement, Kenya 2017. BMC Public Health (2020) 20(1):474. 10.1186/s12889-020-8401-x 32276622 PMC7146951

[33] Pinchoff J Kraus-Perrotta C Austrian K Tidwell JB Abuya T Mwanga D Mobility Patterns during COVID-19 Travel Restrictions in Nairobi Urban Informal Settlements: Who is Leaving Home and Why. J Urban Health (2021) 98:211–21. 10.1007/s11524-020-00507-w 33533010 PMC7852483

[34] Ahorsu DK Lin CY Imani V Saffari M Griffiths MD Pakpour AH The Fear of COVID-19 Scale: Development and Initial Validation. Int J Ment Health Addict (2020) 20:1537–45. 10.1007/s11469-020-00270-8 32226353 PMC7100496

[35] Sotomayor-Beltran C Matta-Solis H Perez-Siguas R Matta-Solis E Matta-Zamudio L. Fear of COVID-19 Among Peruvian People Living in Disadvantaged Communities: A Cross-Sectional Study. Clin Pract Epidemiol Ment Health (2021) 17(1):19–25. 10.2174/1745017902117010019 34040650 PMC8097400

[36] Tanveer R Tarundeep S Sugandhi S Jitender K Dhanajayan G Shubh M Prevalence of Depression and Anxiety during the COVID-19 Pandemic Among the Residents of an Urban Slum in North India. J Neurosci Rural Pract (2021) 12(1):153–8. 10.1055/s-0040-1721623 33531775 PMC7846337

[37] Allande-Cussó R Linares Manrique M Gómez-Salgado J Romero Ruiz A Romero-Martín M García-Iglesias JJ Anxiety and Fear Related to Coronavirus Disease 2019 Assessment in the Spanish Population: A Cross-Sectional Study. Sci Prog (2021) 104(3):003685042110381. 10.1177/00368504211038191 PMC10361589 34435895

[38] Sharma SV Chuang R Rushing M Naylor B Ranjit N Pomeroy M Social Determinants of Health-Related Needs during COVID-19 Among Low-Income Households with Children. Prev Chronic Dis (2020) 17(E119):E119. 10.5888/pcd17.200322 33006541 PMC7553207

