# Corrigendum: Social Determinants of Health in the COVID-19 Pandemic Context of the Migrant Population Living in Settlements in Spain

**DOI:** 10.3389/ijph.2024.1607688

**Published:** 2024-07-09

**Authors:** Regina Allande-Cussó, Juan Jesús García-Iglesias, Rosario Miranda-Plata, Rocío Pichardo-Hexamer, Carlos Ruiz-Frutos, Juan Gómez-Salgado

**Affiliations:** ^1^ Department of Nursing, Faculty of Nursing, Podiatry and Physiotherapy, University of Seville, Seville, Spain; ^2^ Department of Sociology, Social Work and Public Health, Faculty of Labour Sciences, University of Huelva, Huelva, Spain; ^3^ Spanish Red Cross of Huelva, Huelva, Spain; ^4^ Safety and Health Postgraduate Programme, Universidad Espíritu Santo, Guayaquil, Ecuador

**Keywords:** public health, COVID-19, social determinants of health, migrants, psychological distress, fear, settlements, work engagement

In the published article, there was an error in **Affiliation** 2.

Instead of “Department of Sociology, Social Work and Public Health, Faculty of Labour Sciences, Huelva, Spain,” it should be “Department of Sociology, Social Work and Public Health, Faculty of Labour Sciences, University of Huelva, Huelva, Spain.”

In the published article, there was an error in **Affiliation** 4.

Instead of “Safety and Health Postgraduate Programme, Guayaquil, Ecuador,” it should be “Safety and Health Postgraduate Programme, Universidad Espíritu Santo, Guayaquil, Ecuador.”

In the published article, there was an error regarding the **Affiliations** of Juan Gómez-Salgado.

As well as having affiliation 4 (“Safety and Health Postgraduate Programme, Universidad Espíritu Santo, Guayaquil, Ecuador”), they should also have affiliation 2 (“Department of Sociology, Social Work and Public Health, Faculty of Labour Sciences, University of Huelva, Huelva, Spain”) instead of affiliation 5. Affiliation 5 was incorrect and has been removed.

The authors apologize for these errors and state that these does not change the scientific conclusions of the article in any way. The original article has been updated.

